# Prediction models for monitoring selenium and its associated heavy-metal accumulation in four kinds of agro-foods in seleniferous area

**DOI:** 10.3389/fnut.2022.990628

**Published:** 2022-09-23

**Authors:** Linshu Jiao, Liuquan Zhang, Yongzhu Zhang, Ran Wang, Xianjin Liu, Baiyi Lu

**Affiliations:** ^1^Jiangsu Key Laboratory for Food Quality and Safety-State Key Laboratory Cultivation Base, Ministry of Science and Technology, Institute of Food Safety and Nutrition, Jiangsu Academy of Agricultural Sciences, Nanjing, China; ^2^Key Laboratory For Quality Evaluation and Health Benefit of Agro-Products of Ministry of Agriculture and Rural Affairs, College of Biosystems Engineering and Food Science, Key Laboratory for Quality and Safety Risk Assessment of Agro-Products Storage and Preservation of Ministry of Agriculture and Rural Affairs, Zhejiang University, Hangzhou, China

**Keywords:** Se-rich agro-food, selenium, metals, metals accumulation, prediction model

## Abstract

Se-rich agro-foods are effective Se supplements for Se-deficient people, but the associated metals have potential risks to human health. Factors affecting the accumulation of Se and its associated metals in Se-rich agro-foods were obscure, and the prediction models for the accumulation of Se and its associated metals have not been established. In this study, 661 samples of Se-rich rice, garlic, black fungus, and eggs, four typical Se-rich agro-foods in China, and soil, matrix, feed, irrigation, and feeding water were collected and analyzed. The major associated metal for Se-rich rice and garlic was Cd, and that for Se-rich black fungus and egg was Cr. Se and its associated metal contents in Se-rich agro-foods were positively correlated with Se and metal contents in soil, matrix, feed, and matrix organic contents. The Se and Cd contents in Se-rich rice grain and garlic were positively and negatively correlated with soil pH, respectively. Eight models for predicting the content of Se and its main associated metals in Se-rich rice, garlic, black fungus, and eggs were established by multiple linear regression. The accuracy of the constructed models was further validated with blind samples. In summary, this study revealed the main associated metals, factors, and prediction models for Se and metal accumulation in four kinds of Se-rich agro-foods, thus helpful in producing high-quality and healthy Se-rich.

## Introduction

Selenium (Se) has been listed as one of the essential micronutrients by the World Health Organization and the International Health Organization in 1973 (1, 2). Approximately one billion people in the world were estimated to be deficient in Se (3). Severe Se deficiency in the human body could cause myocardial failure, Keshan disease, and Kashin-Beck disease (4–6). China is one of the countries with severe Se deficiency in the world. Se could not be produced by the human body itself; it could only be obtained through diet or pharmaceuticals (7). Qualified Se-rich agro-foods have the characteristics of “safety, high quality, and health,” and they could provide a convenient method to replenish Se in Se-deficient people (8). For example, people in Se-deficient areas, such as Finland, the United Kingdom, Australia, and New Zealand, have used Se-rich agricultural products to increase dietary Se intake to meet the daily Se needs of the human body (9–11).

However, the distribution of Se in nature is uneven and generally exists in a dispersed state. Se often coexists with metals, forming Se-Hg ore, Se-Cu ore, Se-Pb ore, etc., resulting in Se in the natural environment often associated with heavy metals such as Cd, Hg, and Pb (12–14). Moreover, with the development of industrialization and urbanization, sewage irrigation, agricultural materials (such as chemical fertilizers, pesticides, and plastic film) application and solid waste stacking are more serious than before, which may also cause heavy metal pollution in soil (15, 16). Considering the association relationship between Se and heavy metals, the soil heavy metal pollution in Se-rich areas is normally more serious than that in non-Se-rich areas. Thus, agro-foods grown in Se-rich areas could enrich not only Se but also heavy metals, which makes heavy-metal assessment of the Se-rich soil essential before crops planting (17).

Rice is a staple food for residents in many areas of China, and its ability to accumulate Se is strong (18). Therefore, Se-rich rice is regarded as a good Se supplement (19). However, previous studies have shown that rice could easily absorb and accumulate heavy metals from soil (20, 21). Li et al. (22) reported that the Cd and Cd were the dominant contaminants in rice grain, and their concentration were highly influenced by the soil pH, soil organic matter (SOM), Fe fraction and cultivar. Considering the existed association phenomenon of Se and metals in Se-rich rice, the intake of Se-rich rice could become a method of exposure to heavy metals for people who are supplementing Se through diet (23). Besides, many other Se-rich agro-products, such as garlic, mushroom, egg, and meat, are threatened by heavy-metal pollution (7). Therefore, screening factors affecting the accumulation of Se and heavy metals and studying the prediction models of accumulation of Se and associated metals in Se-rich agro-foods are of great importance for controlling the transmission of heavy metals in the food chain and accurately assessing human health risks.

Many prediction models of heavy metals in common agro-foods were established on the basis of indoor simulation experiments or in soil–crop systems (24–27). Most of these models predicted heavy metal content in crops with heavy-metal content in soil and the physical and chemical properties of soil (28, 29). For example, Xu et al. (30) constructed the prediction model of Pb accumulation in rice grain, found that factors containing content of total Pb, clay and SOM performed well in predicting the Pb content in grain. The migration of heavy metals in the soil–crop system not only depends on the content of heavy metals in soil but also the soil physical and chemical properties, which affect the distribution of heavy metals in soil and control the solid-liquid phase distribution of heavy metals, thus affecting the absorption of heavy metals by crops (26, 31). Regression models of loquat trace element concentrations showed that under specific soil condition, the accumulation of Cd, As, and Pb of loquat fruit were controlled by the Ca concentration, metal fraction and Fe content in soil, respectively (32). Similarly, according to the multiple linear regression models constructed by Shi et al. (33), the Pb accumulation in pepper could be quantitatively predicted by soil Pb content, pH and soil cation exchange capacity. Therefore, to improve the accuracy of the prediction results when predicting Se and heavy metal contents in Se-rich agro-foods, metal contents in soil, and soil properties, such as pH and the content of SOM, could be considered as evaluation indicators. At present, the prediction models for the distribution of Se and associated metals in Se-rich agro-foods have not been reported.

In this study, representative main Se-rich producing areas in China were selected to investigate and collect Se-rich rice, eggs, black fungus, and garlic samples and Se-rich soil, matrix, feed, irrigation, and feeding water samples. This study aimed to analyze the distribution of Se and its associated metals in agro-foods, soil, matrix, feed, and water; to screen the main types of associated metals present in Se-rich rice, eggs, black fungus, and garlic in China; and to establish and verify the prediction of Se and its associated metals in these Se-rich agro-foods.

## Materials and methods

### Sample collection

#### Selenium-rich rice sample collection

The collection of samples associated with Se-rich rice has been reported in the author’s previous work (23). In total, 182 samples of Se-rich rice seeds, Se-rich soil, and water were collected, with at least 1 kg for each sample.

#### Selenium-rich garlic sample collection

Selenium-rich garlic, soil, and water used for Se-rich garlic cultivation were collected at the garlic maturity period from May to December in 2019. The sampling sites are distributed in five representative Se-rich garlic production areas (Jinan, Taoyuan, Hailun, Haidong, and Enshi) in China. At least three large-scale Se-rich garlic-producing bases at each area were selected for sample collection, and at least five set of samples (Se-rich garlic, soil, and irrigation water) were collected at each base. In total, 157 samples of Se-rich garlic, Se-rich soil, and water were collected, with at least 1 kg for each sample.

#### Selenium-rich black fungus sample collection

Selenium-rich black fungus and the matrix and water used for Se-rich black fungus cultivation were collected from May to November in 2019. The sampling sites are distributed in five representative Se-rich black fungus production areas (Ankang, Shitai, Fengcheng, Jiamusi, and Enshi) in China. At least three large-scale Se-rich black fungus-producing bases at each area were selected for sample collection, and at least five set of samples (Se-rich black fungus, matrix, and water) were collected at each base. In total, 193 samples of Se-rich black fungus, Se-rich matrix, and water were collected, with at least 1 kg for each sample.

#### Selenium-rich egg sample collection

**FIGURE 1:**
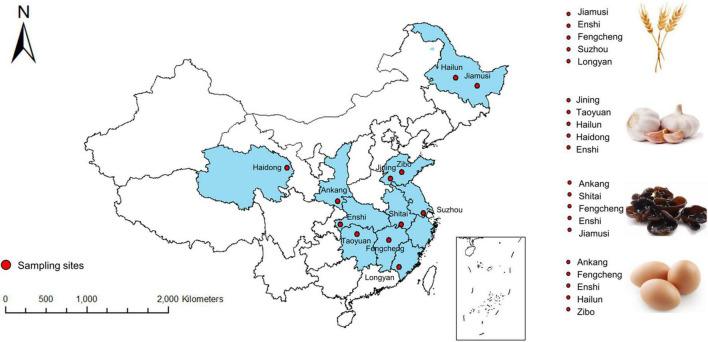
Distribution of sampling sites.

Selenium-rich eggs, the feed and water used for Se-rich egg production were collected from May to October in 2019. The sampling sites are distributed in five representative Se-rich egg production areas (Ankang, Fengcheng, Hailun, Zibo, and Enshi) in China. At least three large-scale Se-rich egg-producing bases at each area were selected for sample collection, and at least five set of samples (Se-rich egg, feed, and water) were collected at each base. In total, 129 samples of Se-rich eggs, Se-rich feed, and water were collected, with at least 1 kg for each sample. Figure 1 presents the sampling sites.

### Sample pretreatment

The collected rice seeds were washed with deionized water and dried in an oven at 60–70°C until reaching a constant weight. The seeds were dehulled to obtain rice grain and then grounded into fine powder. The collected garlics were washed with deionized water and broken into vegetable puree with liquid nitrogen. The dried black fungus was grounded into fine powder. The eggs were washed with deionized water. The egg samples were stored at 4°C, and the rice, garlic, and black fungus samples were stored at −20°C until they were used for further analysis.

Stones and plant tissues were removed from the collected soil samples. The soil was smashed and passed through a 40-mesh sieve after air drying. Then, the sieved soil was grounded and passed through a 100-mesh sieve. The matrix and feed samples were grounded and passed through a 20-mesh sieve after air-drying. The sieved samples of matrix and feed were then passed through a 100-mesh sieve after grounding again. The powder of soil, matrix, and feed was stored in a dry environment at room temperature and used for further analysis.

The collected water, including the irrigation water and feeding water, was filtered with a 0.45 μm filter membrane and injected into the sampling bottle. HNO_3_ was added to the sampling bottle to acidify the water (pH < 2) to fix the heavy-metal elements in the water sample. The pretreated water was stored at 4°C and used for further analysis.

### Sample analysis

#### Analysis of contents of selenium and heavy metals

The contents of total Se and its associated metals, including Cr, Hg, Zn, Cd, Pb, and As, in all samples were analyzed by inductively coupled plasma mass spectrometry (ICP-MS). The population reference intakes (PRIs), tolerable upper intake levels (ULs), provisional tolerated daily intakes (PTDIs) and maximum residue limit values of these elements are listed in Table 1. The detailed procedures and parameters for microwave digestion and ICP-MS referred to one of the authors’ previous studies (7).

**TABLE 1:** PRIs and ULs of selenium and PTDIs and maximum residue limit in agro-foods of potential associated metals.

Elements	PRI (μg/day)			UL (μg/day)			PTDI (μg/kg bw/day)	Maximum residue limit in agro-foods (mg/kg)		
				
	China	WTO/FAO	EFSA	China	WTO/FAO	EFSA	JECFA	China	EU	CAC
Se	60	34 men 26 women	70	400	–	300	–	–	–	–
Zn	12500 men 7500 women	3000–9800	7500–12700	40000	14000	16300	1000	–	–	–
Cd	–	–	–	–	–	–	0.83 (25 μg/kg bw/month)	0.2 rice 0.1 garlic 0.5 black fungus 0.05 egg	0.2 rice 0.1 garlic 1.0 black fungus	0.4 rice 0.05 garlic
Cr	30		–	–	–	–	0.957 (6.7 μg/kg⋅bw/ month)	1.0 rice 0.5 garlic	–	–
As	–	–	–	–	–	–	Not possible to establish a new PTWI that would be considered health protective	0.2 rice 0.5 garlic 0.5 black fungus	–	0.2 rice
Pb	–	–	–	–	–	–	Not possible to establish a new PTWI that would be considered health protective	0.2 rice 0.1 garlic 1.0 black fungus 0.2 egg	0.2 rice 0.1 garlic	0.2 rice 0.1 garlic
Hg		–	–	–	–	–	4	0.02 rice 0.01 garlic 0.1 black fungus 0.05 egg	–	–

PRI, population reference intake; UL, tolerable upper intake levels; PTDI, provisional tolerated daily intake.

Standard solutions purchased from Research Institute of Beijing North Weiye Metrology Technology^1^ were used for calibration. The product numbers of these standard solutions were as follows: Se, GBW(E)080136; Cd, GBW(E)080005; Hg, GBW(E)082530; As, GBW08611; Pb, GBW08619; Cr, GBW08614; and Zn, GSB 04-1761-2004. When the deviation of the test was less than 5%, it was considered a qualified method.

#### pH and organic matter content determination

Sieved soil was mixed with distilled water at the soil–water ratio of 1:5 (w/v), and the pH of the turbid liquid was measured by a pH meter.

Soil organic matter (SOM) content and matrix organic matter (MOM) contents of the samples were measured using an external heating method (34).

### Statistical analysis

Excel 2019 was used for data processing and standard error calculation. SPSS 22.0 was used for statistical analysis, correlation analysis, significant difference analysis, one-way ANOVA, regression analysis, and principal component analysis. The contents of Se and its potential associated metals in the samples were calculated using the @risk 7.0 software, and the final results were shown as fitted values. The metal was defined as associated metal when it showed a very significant positive correlation with Se in the samples, and the correlation coefficient *r*-value > 0.5. The establishment and validation of the prediction model were analyzed using software, such as EViews 10.0 and SPSS 22.0. Python, Origin 2020, and Cytoscape were used for drawing.

## Results

### Screening of associated metals of selenium in four kinds of selenium-rich agro-foods

**FIGURE 2:**
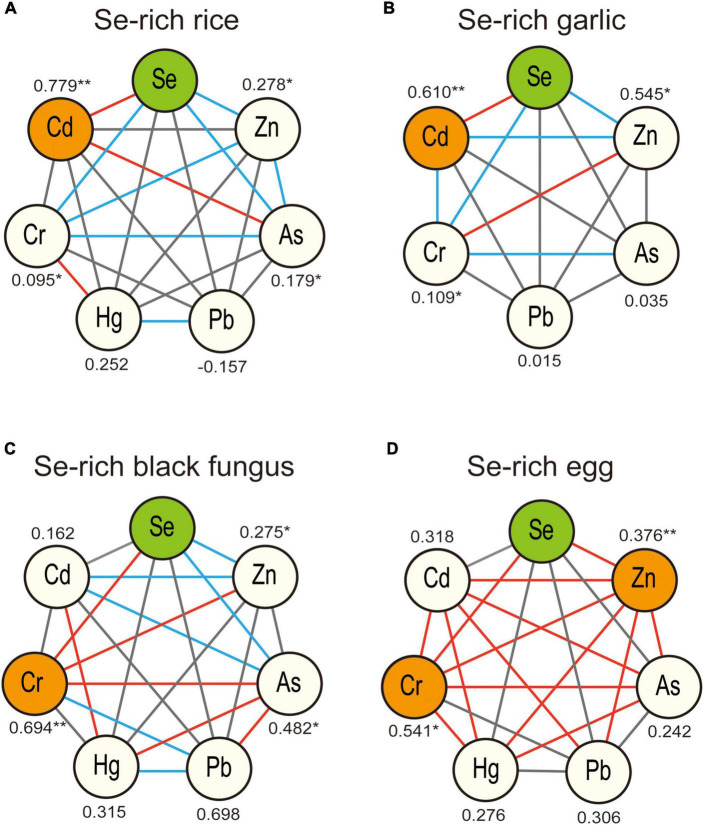
Pearson correlations analysis of Se and potential coexisting elements in Se-rich rice **(A)**, garlic **(B)**, black fungus **(C)**, and egg **(D)**. **Represents for highly significant correlation, *p* < 0.01, *represents for significant correlation, *p* < 0.05. The connections between the nodes represent for the significance of the correlation between different elements, solid red line represents for highly significant correlation, *p* < 0.01, solid blue line represents for significant correlation, *p* < 0.05.

The Pearson correlation coefficients between Se and the six analyzed metals, including Cr, Hg, Zn, Cd, Pb, and As, in Se-rich rice, garlic, black fungus, and egg were calculated to screen which metals were associated with Se. The results are shown in Figure 2, and the detailed correlation coefficients (*r*-value) are listed in Supplementary Table 1.

In Se-rich rice, Se showed highly significant positive correlation with Cd (*r*-value = 0.779, Figure 2A). Se content was also significantly correlated with the contents of Cr, As, and Zn, but the correlations were weak (*r*-value <0.3). In Se-rich garlic, Se was correlated with Cd, Zn, and Cr, and the correlation between Se and Cd (*r*-value = 0.610, Figure 2B) was the strongest. In Se-rich black fungus, Se was significantly correlated with Cr, As, and Zn, with the strongest correlation observed between Se and Cr (*r*-value = 0.694, Figure 2C). Some significant associations were also observed between metals. For example, Cd and Hg was highly associated (*r*-value = 0.706), and As was associated with Cd, Cr, Hg, and Pb. In Se-rich egg, Se was significantly correlated with Cr and Zn. The correlation between Se and Cr (*r*-value = 0.541, Figure 2D) was stronger than that between Se and Zn (*r*-value = 0.376). Some metals were strongly correlated. For example, the *r*-values between Cd and Cr, Cd and Hg, Cr and Hg, Cr and As, and Pb and Zn were all higher than 0.5.

### Screening of factors influencing the accumulation of selenium and its main associated metal in four kinds of selenium-rich agro-foods

Scatter plot and regression analysis were performed to reveal the main factor affecting the accumulation of Se and its main associated metal in Se-rich agro-foods.

**FIGURE 3:**
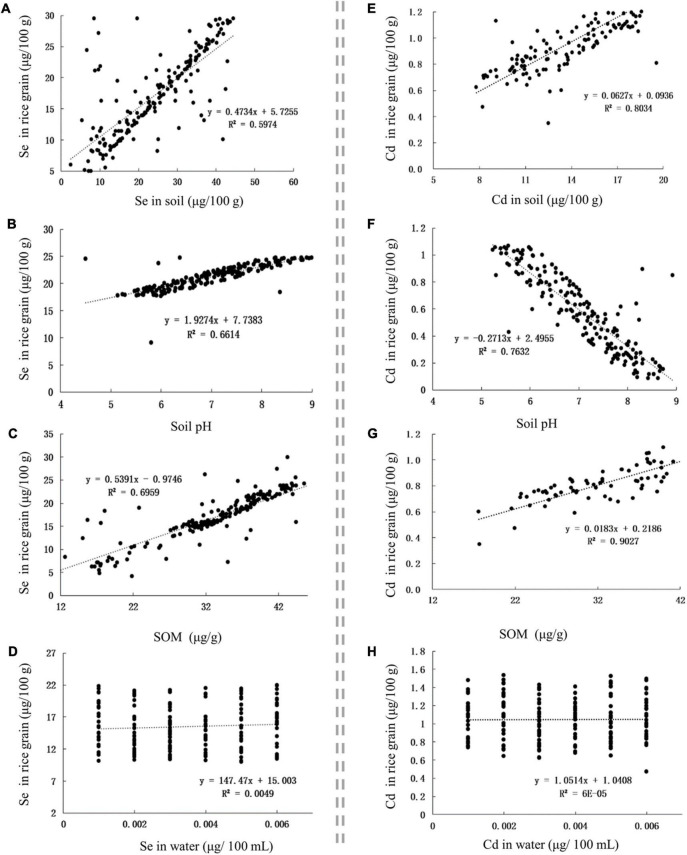
The influence of factors on Se and Cd contents in rice grain. **(A–D)** The influence of Se content in soil, soil pH, SOM, and Se content in water on Se content in rice grain, respectively. **(E–H)** The influence of Cd content in soil, soil pH, SOM, and Cd content in water on Cd content in rice grain, respectively.

The Se content in Se-rich rice grain was positively correlated with the Se content in soil, soil pH, and SOM, with corresponding *r*-values of 0.7729, 0.8133, and 0.8342, respectively (Figures 3A–C). The Cd content in Se-rich rice grain was positively correlated with the Cd content in the soil and SOM but negatively correlated with the soil pH, with corresponding *r*-values of 0.8963, 0.9501, and −0.8736, respectively (Figures 3E–G). The Se and Cd contents in Se-rich rice were not significantly correlated with those in irrigation water (Figures 3D,H).

**FIGURE 4:**
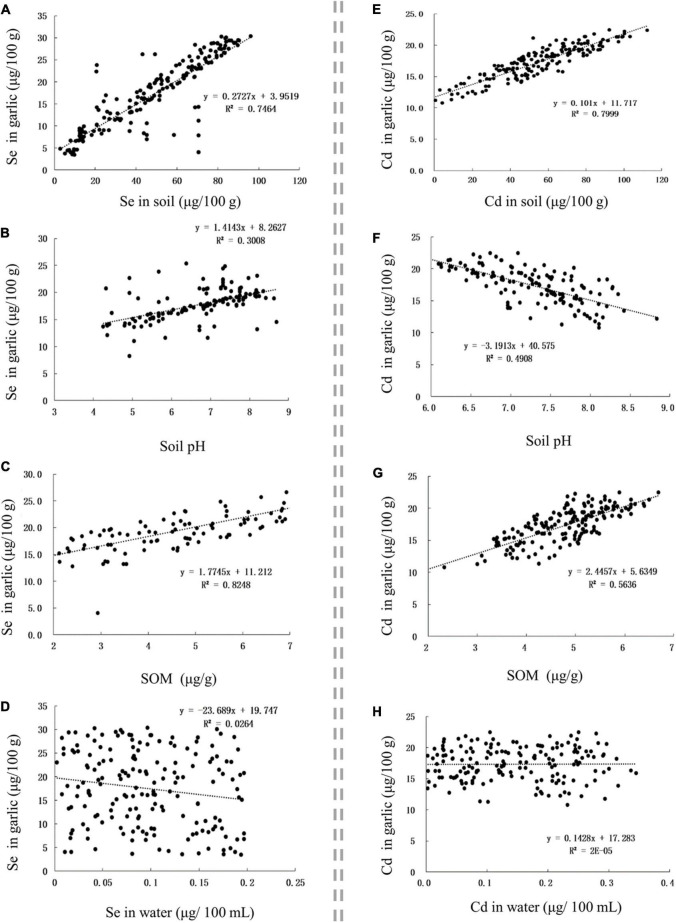
The influence of factors on Se and Cd contents in garlic. **(A–D)** The influence of Se content in soil, soil pH, SOM, and Se content in water on Se content in garlic, respectively. **(E–H)** The influence of Cd content in soil, soil pH, SOM, and Cd content in water on Cd content in garlic, respectively.

The Se content in Se-rich garlic was positively correlated with the Se content in soil, soil pH, and SOM, with corresponding *r*-values of 0.8639, 0.5557, and 0.6113, respectively (Figures 4A–C). The Cd content in Se-rich garlic was positively correlated with the Cd content in soil and SOM but was negatively correlated with soil pH, with corresponding *r*-values of 0.8944, 0.7507, and −0.7006, respectively (Figures 4E–G). The Se and Cd contents in Se-rich garlic were not significantly correlated with those in irrigation water (Figures 4D,H).

**FIGURE 5:**
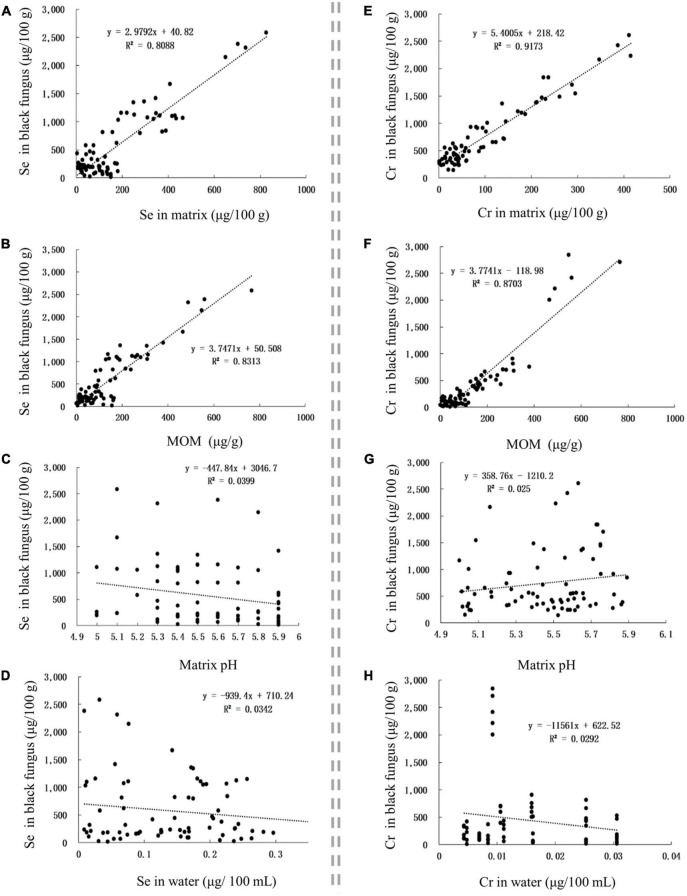
The influence of factors on Se and Cr contents in black fungus. **(A–D)** The influence of Se content in matrix, MOM, matrix pH, and Se content in water on Se content in black fungus, respectively. **(E–H)**, The influence of Cr content in matrix, MOM, matrix pH, and Cr content in water on Cr content in black fungus, respectively.

The Se content in Se-rich black fungus was positively correlated with the Se content in the matrix and MOM, with corresponding *r*-values of 0.8993 and 0.9118, respectively (Figures 5A,B). The Cr content in Se-rich black fungus was positively correlated with the Cr content in the matrix and MOM, with corresponding *r*-values of 0.7879 and 0.9329, respectively (Figures 5E,F). The Se and Cr contents in Se-rich black fungus were not significantly correlated with those in irrigation water nor the pH of the matrix (Figures 5C,D,G,H).

**FIGURE 6:**
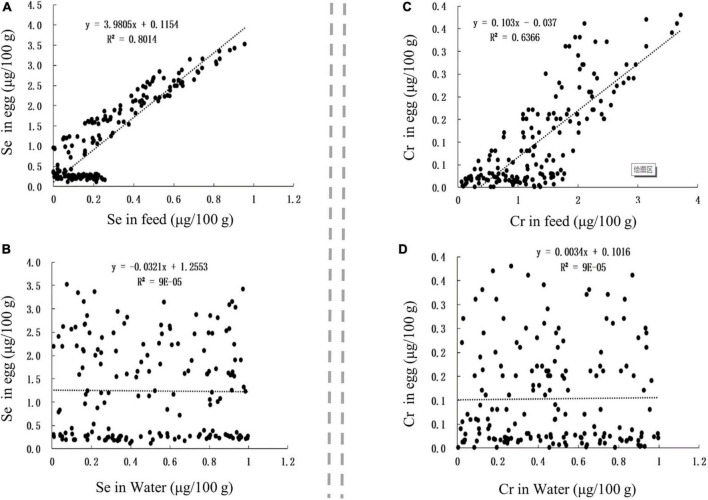
The influence of factors on Se and Cr contents in egg. **(A,B)** The influence of Se content in feed and Se content in water on Se content in egg, respectively. **(C,D)** The influence of Cr content in feed and Cr content in water on Cr content in egg, respectively.

The Se and Cr contents in Se-rich egg were positively correlated with those in the feed (Figures 6A,C), with corresponding *r*-values of 0.8952 and 0.7979, respectively. The Se and Cr contents in Se-rich egg were not significantly correlated with those in feeding water (Figures 6B,D).

### Construction and validation of prediction models for the accumulation of selenium and Cd in selenium-rich rice

Based on the analysis described above, the accumulation of Se in Se-rich rice grains (Se_rice_) was mainly associated with the total amount of Se in the soil (Se_soil_), soil pH (pH_soil_), and SOM. Therefore, the influence of Se_soil_, pH_soil_, and SOM on Se_rice_ was modeled using three-factor multiple linear regression. Two models were established as follows: model I, Se_rice_ = α + β_1_⋅Se_soil_ + β_2_⋅pH_soil_ + β_3_⋅SOM; model I, lg (Se_rice_) = α + β_1_⋅lg(Se_soil_) + β_2_⋅lg(pH_soil_) + β_3_⋅lg(SOM). The parameters of these two models are presented in Table 2. The *R*^2^ and adjusted *R*^2^ (*R*^2^-adj) of these two prediction models were all close to 0.9, indicating that the two models had a high degree of fitting and were valid. Although the *R*^2^ and *R*^2^-adj in model I were slightly bigger than those in model II, the confidence of the lg(pH_soil_) and constant α of model II were much higher than those of the pH_soil_ and constant α of model II. Besides, the sample variance of model II was much smaller than that of model I. Therefore, model II is more suitable to predict the Se content in Se-rich rice, and the equation was as follows: lg(Se_rice_) = −0.1983 + 0.1022 lg(Se_soil_) + 0.1697 lg(pH_soil_) + 0.7545 lg(SOM).

**TABLE 2:** Prediction models of Se and Cd accumulation in Se-rich rice grain.

Model	Variate	Coefficient	*R* ^2^	*R*^2^_adj	T statistic	*P*	Sample variance
Model I*CPSTABLEENTER*Se_rice_	Se_soil_	0.0958	0.8926	0.8944	2.7639	0	3.7724
	pH_soil_	0.2204			2.1448	0.0333	
	SOM	0.0404			17.5977	0.0012	
	C	−0.07635			−0.1307	0.8962	
Model II*CPSTABLEENTER*Se_rice_	lg(Se_soil_)	0.1022	0.8880	0.8898	4.7548	0	0.1037
	lg(pH_soil_)	0.1697			3.5706	0.0005	
	lg(SOM)	0.7545			16.5594	0.0001	
	C	−0.1983			−4.4942	0	
Model I Cd_rice_	Cd_soil_	0.0198	0.5857	0.5787	10.8973	0.0003	0.0542
	pH_soil_	−0.0033			−1.0141	0.3119	
	SOM	0.0008			5.6177	0.0002	
	C	0.0160			3.2413	0.0001	
Model II*CPSTABLEENTER*Cd_rice_	lg(Cd_soil_)	0.8605	0.5650	0.5577	10.6695	0.0001	0.0463
	lg(pH_soil_)	−0.1695			−2.5123	0.0129	
	lg(SOM)	0.3581			5.1465	0.0002	
	C	−0.1748			−1.5140	0.1318	

The accumulation of Cd in Se-rich rice grains (Cd_rice_) was mainly associated with the total amount of Cd in the soil (Cd_soil_), pH_soil_, and SOM. Therefore, the influence of Cd_soil_, pH_soil_, and SOM for Cd_rice_ was also modeled using three-factor multiple linear regression. Two models were established as follows: model I, Cd_rice_ = α + β_1_⋅Cd_soil_ + β_2_⋅pH_soil_ + β_3_⋅SOM; model II, lg (Cd_rice_) = α + β_1_⋅lg(Cd_soil_) + β_2_⋅lg(pH_soil_) + β_3_⋅lg(SOM). The parameters of these two models are also presented in Table 2. The *R*^2^ and *R*^2^-adj of these two prediction models were close to 0.6. The confidence of pH_soil_ of model I was 0.3 > 0.05, while the Cd content in Se-rich rice was significantly and negatively correlated with pH_soil_ (Figure 3F). Therefore, model II is more suitable to predict the Cd content in Se-rich rice, and the equation was as follows: lg(Cd_rice_) = −0.1748 + 0.8605 lg(Cd_soil_)−0.1695 lg(pH_soil_) + 0.3581 lg(SOM).

The content of Se and its associated metals in blind samples were analyzed to verify the accuracy of the constructed model for predicting Se and its main associated metals in Se-rich agro-foods, and then correlation and residual analyses were performed on the measured and predicted values.

**FIGURE 7:**
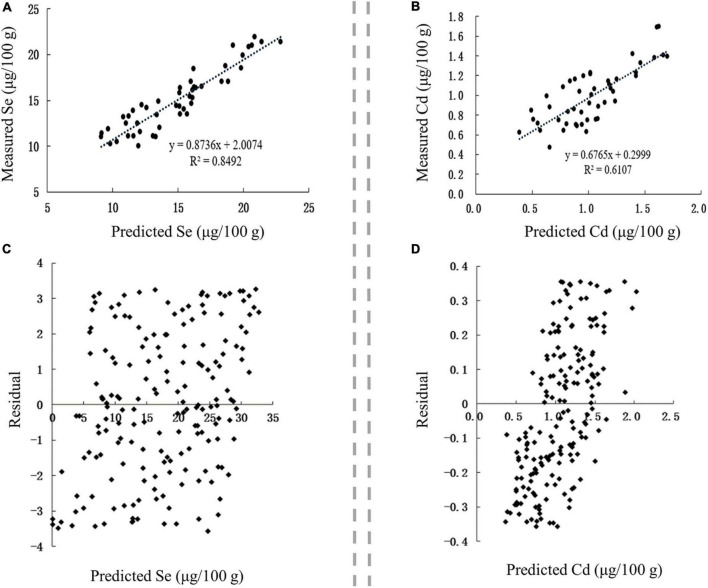
Validation of prediction models in Se-rich rice grain. **(A,B)** Correlation between the fitted and measured Se and Cd contents, respectively. **(C,D)** Residual analysis of the fitted and measured Se and Cd contents, respectively.

The correlation between the predicted and measured values of Se and Cd in Se-rich rice was extremely significant (*p* < 0.01), and the corresponding *r*-values were 0.9215 and 0.7815, respectively (Figures 7A,B). This result indicated that the content of Se and Cd in the soil, soil pH, and SOM could well predict the accumulation of Se and Cd in Se-rich rice. The linear regression equations between the measured values (Se_m_ and Cd_m_) and predicted values (Se_p_ and Cd_p_) were as follows: Se_m_ = 0.8736 Se_p_ + 2.0074 and Cd_m_ = 0.6765 Cd_p_ + 0.2999. The residual distribution of the models was analyzed to further investigate their rationality. The residuals of Se and Cd contents were evenly distributed on both sides of the *Y* = 0 axis and concentrated in ± 4 and ± 0.4, respectively (Figures 7C,D). Collectively, the established models for predicting Se and Cd contents in Se-rich rice were reasonable and reliable.

### Construction and validation of prediction models for the accumulation of selenium and Cd in selenium-rich garlic

The influence of Se_soil_, pH_soil_, and SOM for Se_garlic_ and that of Cd_soil_, pH_soil_, and SOM for Cd_garlic_ were modeled using three-factor multiple linear regression. The processes of modeling and model screening were the same as those for Se_rice_ and Cd_rice_ as described above. The parameters of these models are presented in Table 3.

**TABLE 3:** Prediction models of Se and Cd accumulation in Se-rich garlic.

Model	Variate	Coefficient	*R* ^2^	*R*^2^_adj	T statistic	*P*	Sample variance
Model I*CPSTABLEENTER*Se_garlic_	Se_soil_	0.2649	0.9682	0.9677	72.6167	0	6.7748
	pH_soil_	0.3258			1.9247	0.0559	
	SOM	0.2119			1.9271	0.0556	
	C	5.0587			3.2467	0.0014	
Model II*CPSTABLEENTER*Se_garlic_	lg(Se_soil_)	0.4615	0.9438	0.9428	53.8424	0.0001	0.3425
	lg(pH_soil_)	0.1421			1.8802	0.0617	
	lg(SOM)	0.0590			3.3324	0.0010	
	C	0.9348			5.6488	0	
Model I Cd_garlic_	Cd_soil_	0.0933	0.9889	0.9890	101.5714	0.0003	2.1797
	pH_soil_	−0.4722			−14.2108	0.0001	
	SOM	0.3434			14.9616	0.0002	
	C	15.0199			50.2466	0.0001	
Model II*CPSTABLEENTER*Cd_garlic_	lg(Cd_soil_)	0.1726	0.7385	0.7340	16.64448	0.0001	0.1486
	lg(pH_soil_)	−0.4420			−5.9368	0.0002	
	lg(SOM)	0.0319			1.7658	0.0792	
	C	2.4357			14.5825	0.001	

The *R*^2^ and *R*^2^-adj of the two prediction models for Se_garlic_ were all close to 1, indicating that these two models had a high degree of fitting and were valid. The confidences of variances of models I and II were close, while the coefficient of SOM in model I was much higher than that of lg(SOM) in model II. Therefore, model I is more suitable to predict the Se content in Se-rich garlic, and the equation was as follows: Se_garlic_ = 5.0587 + 0.2649 Se_soil_ + 0.3258 pH_soil_ + 0.2119 SOM.

The *R*^2^ and *R*^2^-adj of model I for Cd_garlic_ were close to 1, much higher than those of model II, indicating that model I had a higher degree of fitting than model II. Besides, the confidences of variances of model I were higher than those of model II, and the confidence of lg(SOM) in model II was 0.079 > 0.05. Thus, model I is more suitable to predict the Cd content in Se-rich garlic, and the equation was as follows: Cd_garlic_ = 15.0199 + 0.0933 Cd_soil_−0.4722 pH_soil_ + 0.3434 SOM.

**FIGURE 8:**
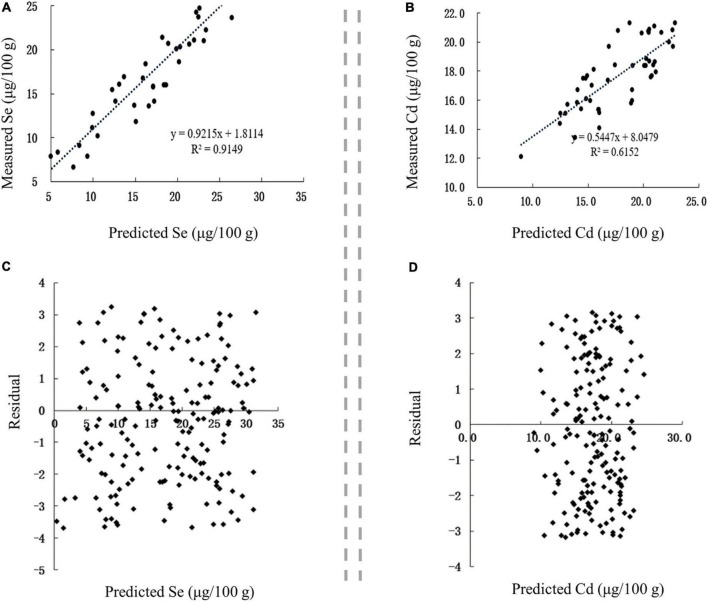
Validation of prediction models in Se-rich garlic. **(A,B)** Correlation between the predicted and measured Se and Cd contents, respectively. **(C,D)** Residual analysis of the fitted and measured Se and Cd contents, respectively.

The correlation between the predicted and measured values of Se and Cd in Se-rich garlic was extremely significant (*p* < 0.01), with corresponding *r*-values of 0.9565 and 0.7843, respectively (Figures 8A,B). The linear regression equations between the measured values (Se_m_ and Cd_m_) and predicted values (Se_p_ and Cd_p_) were as follows: Se_m_ = 0.9215 Se_p_ + 1.8114 and Cd_m_ = 0.5447 Cd_p_+ 8.0479. The residuals of Se and Cd contents were evenly distributed on both sides of the *Y* = 0 axis and concentrated in ± 4 (Figures 8C,D). Collectively, the established models for predicting Se and Cd contents in Se-rich garlic were reasonable and reliable.

Obviously, the prediction models for Se and Cd in rice and garlic showed the relationship with the metal contents, pH, and SOM in soil, which could be attributed to the soil characteristics in seleniferous areas.

### Construction and validation of prediction models for the accumulation of selenium and Cr in selenium-rich black fungus and eggs

The accumulation of Se and Cr in Se-rich black fungus was mainly associated with the total amount of Se and Cr in the matrix and MOM. The prediction models of Se_fungus_ and Cr_fungus_ were established using two-factor multiple linear regression. The parameters of these models are presented in Table 4.

**TABLE 4:** Prediction models of Se and Cr accumulation in Se-rich black fungus.

Model	Variate	Coefficient	*R* ^2^	*R*^2^_adj	T-statistic	*P*	Sample variance
Model I Se_fungus_	Se_matrix_	1.5109	0.8944	0.8914	6.5631	0.0001	602.1944
	MOM	2.1829			7.6430	0.0002	
	C	−1.4197			−0.04337	0.9655	
Model II Se_fungus_	lg(Se_matrix_)	0.2908	0.5599	0.5477	3.3059	0.0015	0.5131
	lg(MOM)	0.5672			6.7161	0.0002	
	C	0.8411			4.5240	0.0001	
Model I Cr_fungus_	Cr_matrix_	3.9994	0.9653	0.9643	11.4330	0.0001	388.0355
	MOM	0.7777			4.7787	0.0002	
	C	−12.1461			−1.0336	0.3048	
Model II Cr_fungus_	lg(Cr_matrix_)	0.6051	0.7148	0.7069	7.5480	0.0001	0.5419
	lg(MOM)	0.3175			3.7705	0.0003	
	C	0.7399			5.7031	0.0002	
							

The *R*^2^ and *R*^2^-adj of model I for Se_fungus_ were close to 0.9, much higher than those of model II, indicating that model I had a higher degree of fitting than model II. Therefore, model I is more suitable to predict the Se content in Se-rich black fungus, and the equation was as follows: Se_fungus_ = −1.4197 + 1.5109 Se_matrix_ + 2.1829 MOM.

The *R*^2^ and *R*^2^-adj of model I for Cr_fungus_ were close to 1, much higher than those of model II, indicating that model I had a higher degree of fitting than model II. Therefore, model I is more suitable to predict the Cr content in Se-rich black fungus, and the equation was as follows: Cr_fungus_ = −12.1461 + 3.9994 Cr_matrix_ + 0.7777 MOM.

**FIGURE 9:**
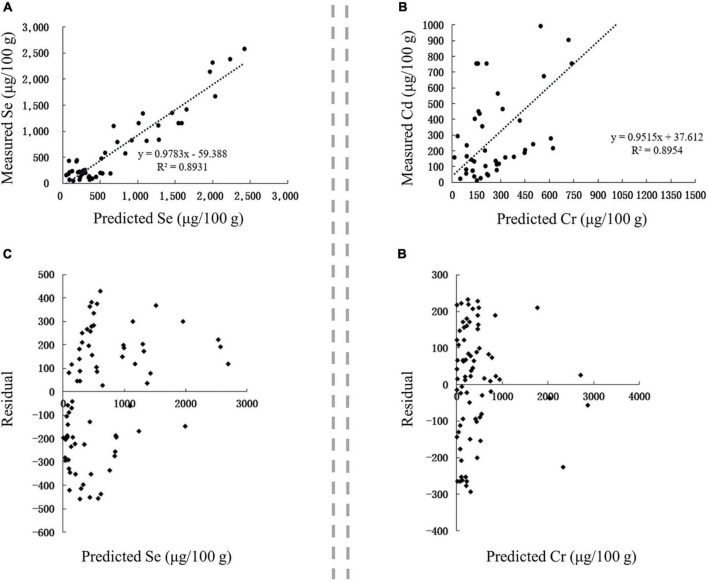
Validation of prediction models in Se-rich black fungus. **(A,B)** Correlation between the predicted and measured Se and Cr contents, respectively. **(C,D)** Residual analysis of the fitted and measured Se and Cr contents, respectively.

The correlation between the predicted and measured values of Se and Cr in Se-rich black fungus was extremely significant (*p* < 0.01), with corresponding *r*-values of 0.9565 and 0.9463, respectively (Figures 9A,B). The linear regression equations between the measured values (Se_m_ and Cr_m_) and predicted values (Se_p_ and Cr_p_) were as follows: Se_m_ = 0.9783 Se_p_59.388 and Cr_m_ = 0.9515 Cr_p_ + 37.612. The residuals of Se and Cr contents were concentrated in ± 500 and ± 300, respectively (Figures 9C,D). Collectively, the established models for predicting Se and Cr content in Se-rich black fungus were reasonable and reliable.

Due to limited variables, the prediction model could be generated as linear regression equation for Se content in Se-rich egg (Se_egg_) on the basis of Se content in the feed (Se_feed_) as follows: Se_egg_ = 0.1154 + 3.9805 Se_feed_, and the *R*^2^ was 0.8014. The linear regression equation for Cr content in Se-rich egg (Cr_egg_) based on Se content in the feed (Cr_feed_) was Cr_egg_ = −0.037 + 0.103 Cr_feed_, and the *R*^2^ was 0.6366.

**FIGURE 10:**
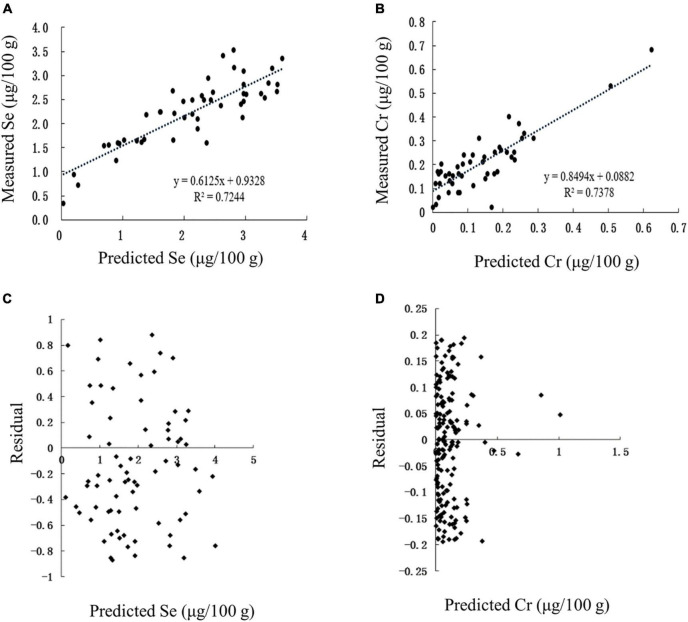
Validation of prediction models in Se-rich egg. **(A,B)** Correlation between the predicted and measured Se and Cr contents, respectively. **(C,D)** Residual analysis of the fitted and measured Se and Cr contents, respectively.

The correlation between the predicted and measured values of Se and Cr in Se-rich egg was extremely significant (*p* < 0.01), with corresponding *r*-values of 0.8511 and 0.8590, respectively (Figures 10A,B). The linear regression equations between the measured values (Se_m_ and Cr_m_) and predicted values (Se_p_ and Cr_p_) were Se_m_ = 0.6125 Se_p_ + 0.9328 and Cr_m_ = 0.8494 Cr_p_ + 0.0882. The residuals of Se and Cr contents were concentrated in ± 1 and ± 0.2, respectively (Figures 10C,D). Collectively, the established models for predicting Se and Cr content in Se-rich egg were reasonable and reliable.

Different from rice and garlic samples, the Se and Cr distribution in black fungus and eggs was mainly influenced by the culture medium and feed instead of soil conditions.

## Discussion

Metals association phenomenon often occurs in seleniferous areas, leading to the associated metals pollution in Se-rich agro-food, and types of related heavy metals differ in various agro-foods (14). Therefore, the association analysis of Se and heavy metals in various agro-foods has important values. In this study, representative main Se-rich producing areas in China were selected to investigate and collect Se-rich rice, eggs, black fungus, and garlic samples and Se-rich soil, matrix, feed, irrigation, and feeding water samples. The typical associated metals with Se in these agro-foods have been screened, the decisive factors for their accumulation have been determined, and the corresponding prediction models also have been constructed.

### Cd association in selenium-rich rice grain and garlic

The most correlated heavy metal has been confirmed to be Cd in both rice grain and garlic in our results. In many natural seleniferous areas, soils are contaminated by heavy metals caused by the weathering of Se-rich shales (35, 36). In particular, Cd has the highest bioavailability, with bioavailable fractions that could reach up to 41.84% of the total Cd in soils; the bioaccumulation factors (BAFs) for crops follow the order Cd > Zn > As > Cu > Ni > Hg > Cr > Pb, making the development of seleniferous area risky (35, 37, 38). In many plants, Se is always applied as an inhibitor for Cd biological toxicity, and Se at specific concentration and valence could efficiently mitigate the oxidative stress induced by Cd, making them coexist in rice (20, 39). The correlation between Se and Cd in rice grain has also been confirmed in the previous work by pot experiment and transcriptome analysis (23). Garlic, which could metabolize inorganic Se into Se amino acids, is an important source for Se amino acid absorption (40, 41). As a plant with good resistance to biotic and abiotic environmental stresses, garlic showed strong resistance to Cd stress, which could be the reason for the Cd accumulation (42).

The accumulation situation of Se and Cd in rice grain and garlic also showed similarity. Their Se content was positively correlated with the Se content in soil, soil pH, and SOM, and their Cd content was positively correlated with Cd content in soil and SOM, and negatively correlated with soil pH. These results were consistent with those of previous studies, that is, the higher the content of Se and Cd in soil, the higher the content of Se and Cd in crops (43). With the increase in soil pH, the transformation of soluble selenate to partially soluble selenite has been inhibited, which further increased the formation of water-soluble Se (44). Meanwhile, the content of water-soluble Cd in soil decreased (45), which may explain why the Se and Cd contents in rice were positively and negatively correlated with soil pH, respectively. Besides, SOM can fix and increase the Se and Cd contents in soil, further enhancing the bioavailability of Se and Cd (46–48).

As reported, the Se content in soil has a high correlation with SOM and pH, and soil Se and pH are the most important parameters that influence Se uptake of crops and vegetables (49, 50). In this study, the Se content in rice grain and garlic was positive influenced by soil pH and SOM, which could also explain the previous results that the application of fertilizer could efficiently increase the Se content in plants (51, 52). Stroud et al. (53) constructed a regression model of Se content in wheat grain, and the total soil Se, extractable soil Se, and extractable soil S were suggested as main controlling parameters for grain Se (Grain Se concentration = −10.32 + 0.1085 × (total soil Se)−1.9 × (extractable soil Se) + 2.515 × (extractable soil S)). The spatial distribution of Se in grain was consistent with that of bioavailable soil Se, which was found to be dominantly influenced by soil pH and SOM (54). However, Gu et al. (37) constructed models for the prediction of BAFs of Se in rice grain and found that BAFs of Se were negatively correlated with soil pH and TOC, which could be related to the difference among sampling sites.

There are more predictive models that have been established for Cd accumulation in rice, focusing on Cd bioavailability influenced by a diversity of soil properties. Soil pH could be the most important factor affecting Cd content in rice grain. In this study, soil pH was negatively correlated with the Cd content in rice grain and garlic. Similarly, Yang et al. (55) reported a Cd prediction model for rice in tin mining area, and the Cd concentration in rice was significantly negative related to the soil pH, and also influenced by CaO, TOC, and Mn in soil. In rice grown both in karst and non-karst areas, the Cd concentration in rice grain was also negative related to the soil pH, and affected by CaO, and Mn in soil (56). The prediction model built by Tang et al. (57), also suggested the Cd content was negatively influenced by soil pH and TFe_2_O_3_ content. The models for Cd prediction in crops exhibit some commonality on the factors. A prediction model of wheat grain Cd was constructed with the variables of soil Cd, soil pH, soil Ca, and coexisting Zn (58). Prediction models of maize and peanut Cd were also determined by TOC, pH, and Mn content in soil (59). Soil Cd content, TOC, and pH values significantly affected the Cd bioaccumulation in sugarcane (57). Interestingly, all of these models exhibited negative relations between Cd accumulation and soil pH, which means the artificial increase of soil pH could be an efficient way to enhance the Se content and decrease the Cd accumulation in the crops at the same time.

The accumulation of many other metals in crops also correlated with the soil properties, such as pH, TOC, cation exchange capacity (CEC), CaO content and Mn content. The predication models showed that Pb content in wheat grain and pepper, and Zn content in rice grain were all negatively influenced by soil pH (33, 60, 61). The BAFs of Cu, Pb, Zn in maize and peanut were negative related with the Mn content in soil (59). Moreover, to improve the application potential, more data covering various varieties, regions and seasons need to be obtained for the construction of prediction models.

### Cr association in selenium-rich black fungus and egg

Edible fungi are good sources of dietary Se, and their Se content could be effectively improved using Se-enriched fermentation culture medium (62). Exogenous Se in organic (Se-enriched yeast) and inorganic (selenite and selenate) forms could be utilized by fungi (63). The addition of dietary Se yeast and selenite supplementation could also increase Se content in laying Longyan duck eggs and Nile tilapia fish tissues (64, 65).

However, edible fungi are detected to have higher heavy metal content compared to crops (66). Therefore, the standards for heavy metal contents in edible fungi were higher than that of crops and egg (Table 1). Mediums containing wheat straw and rice bran are common substrates for edible fungi grown, and they accumulate more heavy metals than corresponding grains. Meanwhile, the raw material pollution, the machinery and equipment exposure during manufacture and environment make the mineral pollution in medium and animal feeds is unavoidable (67). In this study, Cr has been proved as the most associated heavy metal in Se-rich black fungus and egg. Hu et al. (68) reported that Se could significantly reduce the accumulation of Cr in fruiting bodies of black fungus, which may be related to the coexistence of Cr and Se. The health risk assessment of heavy-metal contamination in Se-rich eggs also indicated that Cr was the most correlated heavy metal in Se-rich eggs (69).

Our results showed that Se and Cr content in black fungus were positively correlated with the Se and Cr content in the matrix and MOM. Fungi are sensitive to Se and metals in medium; thus, they could be used to indirectly reflect the pollutants in media, such as water, atmosphere, and soil (70–72). With the addition of increasing levels of selenite, the total Se content in golden needle mushroom (*Flammulina velutipes*) fruiting body also increased (73). The metal intake by fungi is also related to the pH and organic matter contents in the medium (74). Se and Cr accumulation in egg were mainly affected by the Se and Cr content in feed. These results agreed with those of the previous studies, that is, dietary Se supplementation could increase the total egg Se levels, and the total egg Se levels increased with the improvement of the dietary Se supplementation (75, 76). Heavy-metal accumulation in eggs had a positive correlation with the intake of feed contaminated with heavy metals (77), similar to that in fishes and other animals (78). Moreover, a previous study has found that Cr could aggregate more easily than other heavy metals in eggs. When under the same feeding conditions, the Cr content in eggs was higher than that in meat products and liver, which presented higher risks (67). The prediction models of Cr in black fungus and egg could be used for the safety assessment of cultural medium and feeds for Se-rich fungus and eggs. In summary, the common ground of heavy-metal accumulation in rice grain and garlic indicate that the detection and treatment of Cd pollution are essential before the development of Se-rich land resources; and the Cr pollution in mushroom medium and poultry feed should not also be ignored. The prediction model of heavy metals in the agro-foods could be used for the assessment of Se-rich soils, cultural medium and feeds.

## Conclusion

Some metals were associated with Se in Se-rich agro-foods. The main associated metal in Se-rich rice grain and garlic was Cd, and the main associated metal in Se-rich black fungus and egg was Cr. The contents of Se and its associated metal in Se-rich agro-foods was highly and positively correlated with those of the corresponding element in soil, matrix, feed, SOM, and MOM. The Se content in Se-rich rice grain and garlic was also positively correlated with soil pH, whereas the content of the main associated metals in these two agro-foods was negatively correlated with soil pH. The contents of Se and metals in irrigation waters for plants and feeding water for hens had no significant effect on the content of Se and metals in Se-rich agro-foods. Eight models for prediction of Se and its main associated metals in Se-rich rice grain, garlic, black fungus, and egg were established using multiple linear regression analysis. The *R*^2^ and *R*^2^-adj of each model were high. The high accuracy of these models was validated by correlation analysis between the measured values and the predicted values of the blind samples and residual analysis. In this study, the main associated metals and the main factors affecting the accumulation of Se and the main associated metals were revealed. The prediction models in four typical Se-rich agro-foods were also established and validated, providing valuable guidance to produce high-quality and healthy Se-rich agro-foods.

## Data availability statement

The original contributions presented in this study are included in the article/Supplementary material, further inquiries can be directed to the corresponding authors.

## Author contributions

LJ: conceptualization, data curation, investigation, and writing—original draft. LZ: conceptualization, resources, methodology, and data curation. YZ: investigation, software, data curation, and formal analysis. RW and XL: supervision and funding acquisition. BL: supervision, project administration, funding acquisition, and writing—review and editing. All authors contributed to the article and approved the submitted version.

## Footnotes

1
http://www.bzwz.com/
