# Supplementary material for: Prediction models for monitoring selenium and its associated heavy-metal accumulation in four kinds of agro-foods in seleniferous area
Source: Front Nutr. 2022 Sep 23;9:990628. doi: 10.3389/fnut.2022.990628 (PMC9537640; doi:10.3389/fnut.2022.990628)
Supplement: Supplementary file 1 [file Table_1.DOCX]

**Table S1.** Correlation coefficient of selenium and its associated metals in 4 kinds of selenium-rich agro-foods

| Agro-foods | Elements | Se | Cd | Cr | Hg | Pb | As | Zn |
| --- | --- | --- | --- | --- | --- | --- | --- | --- |
| Selenium-rich rice | Se | 1.000 | 0.779** | 0.095* | 0.252 | -0.157 | 0.179* | 0.278* |
|  | Cd | — | 1.000 | -0.098 | -0.092 | 0.129 | 0.392** | 0.023 |
|  | Cr | — | — | 1.000 | 0.192** | 0.072 | 0.196* | 0.130* |
|  | Hg | — | — | — | 1.000 | 0.185* | -0.245** | 0.214 |
|  | Pb | — | — | — | — | 1.000 | 0.120 | -0.153 |
|  | As | — | — | — | — | — | 1.000 | 0.294* |
|  | Zn | — | — | — | — | — | — | 1.000 |
| Selenium-rich garlic | Se | 1.000 | 0.610^**^ | 0.109^*^ | — | 0.015 | 0.035 | 0.545^*^ |
|  | Cd | — | 1.000 | 0.296^*^ | — | -0.029 | -0.061 | 0.559^*^ |
|  | Cr | — | — | 1.000 | — | -0.016 | -0.037 | 0.283^**^ |
|  | Hg | — | — | — | — | — | — | — |
|  | Pb | — | — | — | — | 1.000 | 0.050 | 0.064 |
|  | As | — | — | — | — | — | 1.000 | 0.143 |
|  | Zn | — | — | — | — | — | — | 1.000 |
| Selenium-rich black fungus | Se | 1.000 | 0.162 | 0.694^**^ | 0.315 | 0.698 | 0.482^*^ | 0.275^*^ |
|  | Cd | — | 1.000 | 0.043 | 0.706^**^ | 0.026 | 0.289^*^ | 0.288^*^ |
|  | Cr | — | — | 1.000 | 0.169 | 0.265^*^ | 0.495^**^ | 0.299^**^ |
|  | Hg | — | — | — | 1.000 | 0.284^*^ | 0.545^**^ | 0.086 |
|  | Pb | — | — | — | — | 1.000 | 0.353^**^ | 0.110 |
|  | As | — | — | — | — | — | 1.000 | 0.213 |
|  | Zn | — | — | — | — | — | — | 1.000 |
| Selenium-rich egg | Se | 1.000 | 0.318 | 0.541^**^ | 0.276 | 0.306 | 0.242 | 0.376^**^ |
|  | Cd | — | 1.000 | 0.640^**^ | 0.559^**^ | 0.270^**^ | 0.344^**^ | 0.394^**^ |
|  | Cr | — | — | 1.000 | 0.588^**^ | 0.156 | 0.623^**^ | 0.343^**^ |
|  | Hg | — | — | — | 1.000 | -0.123 | 0.391^**^ | 0.098 |
|  | Pb | — | — | — | — | 1.000 | 0.135 | 0.610^**^ |
|  | As | — | — | — | — | — | 1.000 | 0.418^**^ |
|  | Zn | — | — | — | — | — | — | 1.000 |

** indicates extremely significant correlation; * indicates significant correlation.
